# The CADTH pCODR Expert Review Committee Process Explained. Comment on Rayson et al. Access to Neoadjuvant Pertuzumab for HER2 Positive Breast Cancer in Canada: A Dilemma Increasingly Difficult to Explain. *Curr. Oncol.* 2022, *29*, 9891–9895

**DOI:** 10.3390/curroncol30050380

**Published:** 2023-05-16

**Authors:** Maureen Trudeau, Brent Fraser

**Affiliations:** 1 Sunnybrook Health Sciences Centre, Toronto, ON M4N 3M5, Canada; 2 CADTH, Ottawa, ON K1S 5S8, Canada

CADTH read with interest the commentary published on 16 December 2022, entitled “Access to Neoadjuvant Pertuzumab for HER2 Positive Breast Cancer in Canada: A Dilemma Increasingly Difficult to Explain” [1], which discusses the CADTH reimbursement review of pertuzumab for HER2-positive breast cancer. We noticed a number of inaccuracies in the description of the appraisal process and deliberation by CADTH’s pan-Canadian Oncology Drug Review Expert Review Committee (pERC). We would like to take this opportunity to provide clarification and to inform your readers, authors, and reviewers about the work CADTH does.

CADTH is a not-for-profit organization responsible for providing Canada’s health care decision makers with objective evidence to help make informed decisions about the optimal use of drugs and medical devices in the Canadian health care system. CADTH’s mandate is to deliver evidence-based information on the appropriate use of drugs and other health technologies to ensure that health systems in Canada are sustainable and deliver high value. As part of this work, CADTH delivers nonbinding, evidence-informed reimbursement recommendations and advice to federal, provincial, and territorial public drug plans and provincial cancer agencies, with the exception of those in Quebec. CADTH does not make funding decisions; rather, each public drug plan makes its own decisions based on CADTH’s recommendation and other factors, including the plan’s mandate, jurisdictional priorities, and financial resources.

CADTH uses a health technology assessment (HTA) process to assess the value of a drug or a medical device. HTA is a multidisciplinary process that uses explicit methods to determine the value of a drug or medical device at different points in its life cycle. Its purpose is to inform decision making, and thereby promote an equitable, efficient, and high-quality health system [2].

The authors of the commentary suggest that it is difficult to explain why CADTH issued a “do not reimburse” recommendation for neoadjuvant pertuzumab for HER2-positive breast cancer. We would like to provide clarity by describing how CADTH conducts reimbursement reviews, the information we gather, and the deliberative process used by our expert committees.

In terms of our drug reimbursement review process, a sponsor (typically the pharmaceutical company that manufactures the drug) files an application with CADTH and submits a dossier of evidence, which we use to conduct our HTA. The sponsor’s submission triggers our review, our appraisal of the evidence, and the pERC deliberation. CADTH is committed to our service standard of issuing a draft reimbursement recommendation within 180 days from review initiation. It is important to note that pERC membership includes individuals with expertise in cancer drug therapy, drug evaluation, and drug utilization, as well as patient members who present the patient input at the outset of the deliberations. CADTH reviews the clinical studies and pharmacoeconomic model included in the dossier, along with input from stakeholders including patient groups, clinician groups, clinical experts consulted by CADTH throughout the review, and the public drug plans.

CADTH may include evidence other than randomized control trials (e.g., real-world evidence and observational studies) within the clinical review when it will provide information to fill an evidence gap. Updated clinical trial information can be incorporated into a reimbursement review up until the draft CADTH reports have been sent to the sponsor for comment. CADTH relies on the sponsor during the 180-day review period (the service standard provided to industry) to provide any updated evidence in a timely manner. Furthermore, the onus is on the sponsor to conduct robust studies to demonstrate a drug’s value so that CADTH, other HTA organizations, and decision makers have the information required to make a decision.

If new information becomes available after a final CADTH recommendation is issued, the sponsor has the option of filing a resubmission (for a drug that received a “do not reimburse” recommendation) or reassessment (for a drug that received a recommendation to reimburse with conditions). New evidence submitted must address any evidence gaps identified by pERC. If the new information is determined to address previously identified gaps in the evidence, the file will be placed on the agenda for an upcoming pERC meeting, during which the new information is reviewed and the previous recommendation could be changed or upheld, based on the strength of the new information. CADTH welcomes inquiries regarding resubmissions and reassessments for previously reviewed drugs; however, CADTH does not pursue a sponsor in such circumstances.

We want to emphasize that each dossier submitted is evaluated on its own merit. At pERC, the committee deliberation considers the magnitude of clinical benefit over currently available treatments, the quality of the evidence, the unmet need at the time of review, and the drug’s anticipated place in therapy.

Regarding the drug that is the focus of the commentary, we would like to point out that the goal of neoadjuvant pertuzumab for HER2-positive breast cancer is to cure the cancer. The sponsor submitted two efficacy-focused pivotal trials (four trials were submitted: two were efficacy-focused pivotal trials and two assessed safety and tolerability) with a primary outcome of pathologic complete response (pCR), which is a surrogate outcome. A number of meta-analyses assessed whether pCR is a valid prognostic marker for overall survival or event-free survival in breast cancer. While there is evidence for pCR as a prognostic factor on an individual patient basis, there is not sufficient evidence to identify the magnitude of pCR improvement that predicts long-term prognosis on a trial level. https://www.cadth.ca/sites/default/files/DRR/2022/PC0241-Perjeta.pdf (accessed on 17 January 2023)

Another important clarification is that the pharmacoeconomic model CADTH uses to determine the comparative cost-effectiveness of a drug is one that is submitted to us by the sponsor. CADTH examines the submitted economic model, its parameter inputs, and its assumptions. In order to test the robustness of the model, we conduct a series of reanalyses to test the model function and its assumptions. A pharmacoeconomic analysis alone does not form the basis of a “do not reimburse” recommendation. A recommendation to not reimburse a drug is issued when the clinical benefit, that is at least comparable relative to other treatments reimbursed by public drug plans at the time of the review, has not been demonstrated.

CADTH appreciates the opportunity to provide some insight on the pan-Canadian drug reimbursement review process. We invite your readers to visit cadth.ca/cadth-reimbursement-reviews to learn more about the procedures we follow when conducting our reimbursement reviews.
